# Unitig-centered pan-genome machine learning approach for predicting antibiotic resistance and discovering novel resistance genes in bacterial strains

**DOI:** 10.1016/j.csbj.2024.04.035

**Published:** 2024-04-16

**Authors:** Duyen Thi Do, Ming-Ren Yang, Tran Nam Son Vo, Nguyen Quoc Khanh Le, Yu-Wei Wu

**Affiliations:** a Graduate Institute of Biomedical Informatics, College of Medical Science and Technology, Taipei Medical University, Taipei, Taiwan; b Department of Electrical Engineering, National Taiwan University of Science and Technology, Taipei, Taiwan; c Department of Business Administration, College of Management, Lunghwa University of Science and Technology, Taoyuan City, Taiwan; d Professional Master Program in Artificial Intelligence in Medicine, College of Medicine, Taipei Medical University, Taipei, Taiwan; e Clinical Big Data Research Center, Taipei Medical University Hospital, Taipei, Taiwan; f TMU Research Center for Digestive Medicine, Taipei Medical University, Taipei, Taiwan

**Keywords:** Antimicrobial resistance, Unitig, De Bruijn graph, Feature selection, *Pseudomonas aeruginosa*

## Abstract

In current genomic research, the widely used methods for predicting antimicrobial resistance (AMR) often rely on prior knowledge of known AMR genes or reference genomes. However, these methods have limitations, potentially resulting in imprecise predictions owing to incomplete coverage of AMR mechanisms and genetic variations. To overcome these limitations, we propose a pan-genome-based machine learning approach to advance our understanding of AMR gene repertoires and uncover possible feature sets for precise AMR classification. By building compacted de Brujin graphs (cDBGs) from thousands of genomes and collecting the presence/absence patterns of unique sequences (unitigs) for *Pseudomonas aeruginosa*, we determined that using machine learning models on unitig-centered pan-genomes showed significant promise for accurately predicting the antibiotic resistance or susceptibility of microbial strains. Applying a feature-selection-based machine learning algorithm led to satisfactory predictive performance for the training dataset (with an area under the receiver operating characteristic curve (AUC) of > 0.929) and an independent validation dataset (AUC, approximately 0.77). Furthermore, the selected unitigs revealed previously unidentified resistance genes, allowing for the expansion of the resistance gene repertoire to those that have not previously been described in the literature on antibiotic resistance. These results demonstrate that our proposed unitig-based pan-genome feature set was effective in constructing machine learning predictors that could accurately identify AMR pathogens. Gene sets extracted using this approach may offer valuable insights into expanding known AMR genes and forming new hypotheses to uncover the underlying mechanisms of bacterial AMR.

## Highlights

• Compacted de Bruijn graph is utilized to build unitigs from bacterial genomes.
• A unitig-based feature selection model for antimicrobial resistance prediction.
• *Pseudomonas aeruginosa* antimicrobial resistances can be predicted accurately.
• Genes extracted from selected unitigs reveal key factors in co-resistances.

## Graphical Abstract

**Figure:**
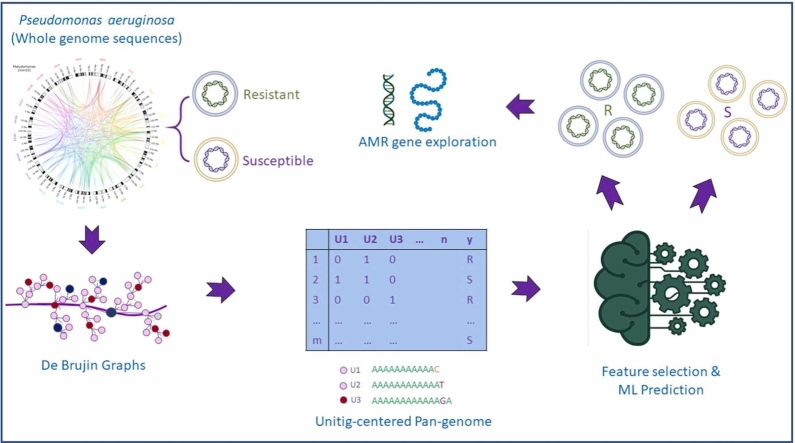

## 1 Introduction

Antimicrobial-resistant (AMR) pathogens are a major concern in modern medicine, where germs can now withstand previously effective drugs. Such pathogens pose significant global public health threats, as they render antimicrobial treatments ineffective and promote the spread of microbial infections. This problem is exacerbated by the fact that bacteria can acquire antibiotic resistance relatively quickly, as demonstrated by the well-known MEGA-plate molecular evolutionary genetics experiment [1], thereby surpassing the pace at which most pharmaceutical companies can create new medications. Identifying the determinants of resistances from whole genome sequencing (WGS) data is a critical step in combating AMR. It allows for the study and comprehension of the biological mechanisms underlying AMR, offering important insights into the development and design of rapid and precise clinical diagnostics for AMR, as well as new antimicrobial drugs.

Owing to technological advances in rapid, high-throughput sequencing, it has become more feasible to directly predict AMR phenotypes from whole-genome data, as the cost of genotyping has declined. To predict AMR from whole-genome genotype data, variations between individual genomes were examined and associated with phenotypes. A commonly used method is a genome-wide association study (GWAS), a research strategy that involves numerical representations of genomic variations to uncover statistical connections with the likelihood of developing a certain disease or characteristics within a population. Such studies have recently shown promising results in identifying the genetic determinants of AMR owing to the availability of bacterial genomes and associated phenotypic data [2], [3], [4], [5], [6]. A typical GWAS uses a statistical significance test to determine the *p*-value for each single-nucleotide polymorphism (SNP) and then establishes a threshold to output only the most significant SNPs. However, this approach has limitations, because the accuracy of the results depends on how well the statistical model corresponds to the actual data distributions. Additionally, different GWAS packages may produce different outcomes, and some may miss certain causative SNPs. Furthermore, *p*-values only demonstrate the presence of a relationship between SNPs and a phenotype and not the strength of that relationship. For this reason, SNPs selected by GWASs may not be accurate predictors; thus, one should not primarily base predictive models on them [7].

In addition, because of the enormous strain-to-strain variances and the fact that information on these variations is mostly based on a single reference genome, utilizing GWASs on bacterial genomes can be challenging. To overcome this limitation, recent studies have sequenced numerous genomes of particular species and extensively studied variations in known AMR genes that confer resistance. By leveraging this knowledge, researchers have successfully created more precise models to determine the phenotypes of various bacterial species [8], [9], [10], [11]. This strategy has been shown to be successful in several cases, because AMR is frequently associated with a single gene or a limited number of variations. However, the main drawback is that novel AMR components may not be identified when concentrating on a limited number of genes with established characteristics, similar to the current detection methods.

Recent studies have increasingly turned to k-mers to enhance bacterial genomic GWASs by addressing the limitations of reference-based methods [12], [13], [14]. K-mer analyses allow the exploration of numerous genetic phenomena, such as SNPs, recombinations, and insertions/deletions, without the need for a reference genome or assumptions regarding causal variants, and can even be performed without the need for assembling genomic sequences. However, k-mers do not reflect biological objects, often necessitating the mapping of numerous short sequences to convert the k-mer-based GWAS results into meaningful genetic insights. Recent studies have suggested that reassembly is significantly related to k-mers to eliminate redundancy and obtain longer marker sequences [14], [15]. Furthermore, the interpretability of k-mer representations often decreases as their flexibility increases, and an optimal method to encode genomic variations in bacterial GWASs is needed [16], [17]. The de Bruijn graph (DBG) GWAS (DBGWAS) was developed to resolve this issue by filling in the gaps between two existing methods: one utilizes SNPs and genes to describe data but lacks the flexibility to account for all genomic variations, whereas the other employs k-mers, which are more adaptable but difficult to interpret [18]. By relying on the capability of compacted DBGs (cDBGs), DBGWAS can capture genetic differences, reduce duplicate information in a particular region, and characterize the genomic context of a k-mer for a specific population. cDBGs are a well-established structure used in genome assembly [19], [20] and variant calling that link overlapping k-mers (short fragments of DNA), resulting in a concise summary of all variations present in a group of genomes.

The goal of this study was to devise an alternative method for GWASs that leverages genomic data and explainable machine learning (ML) techniques to elucidate the AMR gene repertoire and identify key feature sets for precise AMR classification. Unlike GWASs, this data-driven approach requires no preexisting model assumptions and relies exclusively on predictive capabilities to discriminate between AMR determinants. To capture the full genetic diversity within a species and reduce bias stemming from a single reference genome, we incorporated DBGWASs to create a unitig-based pan-genome that encompasses shared sequences among all available strains of a particular bacterium [18]. This was achieved by constructing a cDBG from a collection of all available *P. aeruginosa* genomes downloaded from the Pathosystems Resource Integration Center (PATRIC) [21]. We then extracted and analyzed graph nodes, referred to as unitigs, to identify genetic differences and overlaps among the genomes. Unlike the gene-centric pan-genomic approach, which predominantly hinges on the presence or absence of specific individual genes that offer limited insights, our unitig-centered pan-genome methodology strongly emphasizes genetic sequences. This methodology enabled us to reveal discrete or shared segments of the entire genome, shedding light on genes within the accessory genome and non-coding genes. This approach has played a key role in the development of predictive models to identify genetic factors that affect AMR phenotypes.

By testing the DBGWAS method on pan-genomes constructed from thousands of genomic sequences, our proposed model was determined to be highly effective in predicting AMR genes in *P. aeruginosa.* We also discovered a diverse range of AMR mechanisms and a large number of genes with unknown functions. These findings indicate that the pathogen tends to develop more complex AMR pathways, which compromises the effectiveness of multiple drugs. Additionally, our research revealed novel co-resistance genes that potentially contribute to multidrug resistance (MDR), including mobile genetic elements (MGEs), such as phage proteins, integrases, and transposases, which are consistently prominent among these co-resistance genes. Interestingly, although the type I secretion system (T1SS–T6SS) categories ranked fourth in terms of gene percentages among other virulence factors such as efflux pumps, two-component signaling systems (TCSSs), genes associated with T1SS, T4SS, and T6SS notably emerged as the second most prominent group within the co-resistant gene intersections. This underscores their significant roles in the development of MDR. Additionally, we identified genes, such as *VgrG protein* (T6SS), *VirB4* (T4SS), *VirD4* (T4SS), and large exoproteins involved in heme utilization or adhesion, which indirectly contribute to AMR development. These findings emphasize the need for further research to elucidate the functions and potential roles of these genes in AMR.

## 2 Methods

**Fig. 1:**
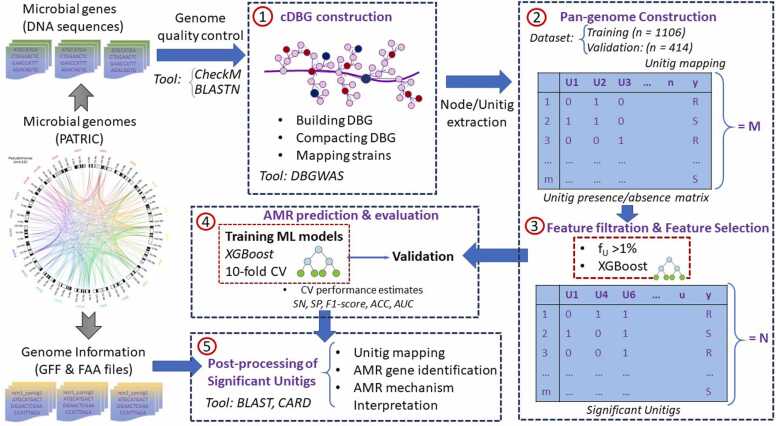
Comprehensive workflow for AMR predictions: from compacted de Bruijn graph (cDBG) construction to feature selection and analysis.

The analytical pipeline of this study, illustrated in Fig. 1, encompasses a comprehensive workflow for AMR prediction using *P. aeruginosa* genomic data. The process began with data collection, in which bacterial data, including DNA sequences, phenotype information, and relevant data, were downloaded. Once the data were collected, a genome quality assessment was conducted using CheckM [22] for genome quality and Basic Local Alignment Search Tool (BLASTN) [23] to rule out possible contamination. This ensured the reliability and quality of the genomic sequences. The next step involved the construction of cDBG from the genomic data using DBGWAS [18]. cDBG facilitated the identification and collection of nodes/unitigs, which were then used to construct a unitig-centered pan-genome. The pan-genome represents the collective genomic content of the *P. aeruginosa* strains. Subsequently, feature filtration and selection were performed to identify the relevant features for AMR prediction. This step helped to determine the genomic characteristics or patterns that were most informative for predicting AMR. The subsequent stage involved training and validating the AMR predictor using both the cross-validation and validation datasets collected in an independent study. In the post-processing step, significant unitigs were mapped back to their respective genomes to explore novel AMR genes and interpret the AMR mechanisms.

## 3 Data collection

*P. aeruginosa* is a prevalent bacterium that causes various types of infections. Their ability to transfer genetic material within and between species makes them highly versatile [24]. The accessory genome of this species plays a vital role in terms of its vast diversity and size, and comprises more than 50% of the known genetic factors responsible for conferring antimicrobial drug resistance [25], [26], [27].

In this study, a model was developed using a specific training dataset and an independent dataset was used for validation. For the training dataset, we retrieved the genomic sequences of *P. aeruginosa* and AMR phenotype (PATRIC_genomes_AMR.txt) from the PATRIC database [21]. Detailed assembly statistics, including the average genome length, minimum contig count, N50 values, and genome status, are provided in Supplementary Table S1. To ensure the reliability and quality of the pan-genome, the completeness and contamination levels of the genomic sequences were first assessed using CheckM v1.1.3 [22] to exclude poor-quality genomes. Additionally, 16 S ribosomal RNA subunit genes from the National Center for Biotechnology Information (NCBI)-listed *P. aeruginosa* reference genome (*P. aeruginosa* PAO1; GenBank accession no. NC_002516.2) were obtained and compared to the downloaded genomes using NCBI BLASTN (parameters: -evalue 1e-10 -max_target_seqs 1) [23]. We retained only those strains for subsequent analysis with a completeness of at least 95%, contamination levels of no more than 5%, and 16 S BLAST identity of at least 99%.

To improve the reliability and generalizability of our research, we gathered an external independent validation dataset from the study by Khaledi et al. [28]. It comprises 414 clinical isolates of *P. aeruginosa* obtained from various clinical and research institutions. After downloading the sequencing reads of the clinical isolates from the NCBI SRA website (https://www.ncbi.nlm.nih.gov/sra), each paired-end read set was trimmed using Trimmomatic v0.39 (with the following parameters: ILLUMINACLIP:TruSeq3-PE. fa:2:30:10 LEADING:20 TRAILING:20 SLIDINGWINDOW:4:15 MINLEN:36) [29] and then assembled using SPAdes v3.13.0 (with --careful options) [30]. The assembly statistics of the 414 isolates from the validation dataset are provided in Supplementary Table S1. Because the susceptibilities of the clinical isolates in the validation dataset were only tested for four antibiotics (tobramycin, ciprofloxacin, ceftazidime, and meropenem), we included only these four drug datasets for validation purposes. Importantly, the validation dataset was intentionally kept separate from the model training process to assess the performance of the model using unseen data. By withholding this dataset during training, we can accurately evaluate the ability of the model to generalize new and unseen samples, thus ensuring its reliability and effectiveness in real-world scenarios.

### 3.1 cDBG construction

**Fig. 2:**
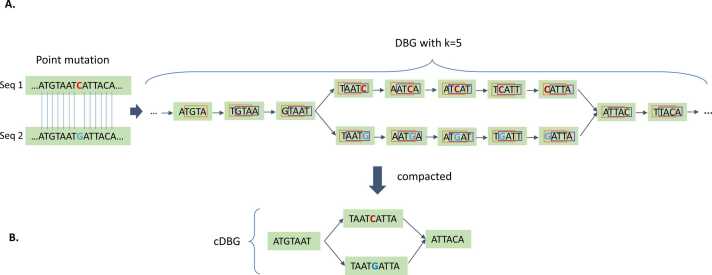
A simple example illustrating the construction of a cDBG for a collection of single-point-mutated sequences. A. A compilation of all k-mers (k = 5) present in two sequences (Seq 1 and 2) was generated. The last k-1 = 4 nucleotides of the first k-mer must match the first four nucleotides of the second k-mer in order to be connected. The bubble pattern denotes the single-nucleotide polymorphism (SNP) from C to G, with each arm of the bubble representing an allele. B. By compacting the linear paths of the graph, a more-concise representation of the DBG is obtained. Compared to the original DBG, which consisted of 15 nodes (k-mers), the cDBG can effectively capture the same variation with only four nodes (unitigs).

DBGs are a type of directed graphs that can effectively capture all information present in a given set of sequences. Each node in the graph represents a distinct k-mer, which is a substring of the genome sequence with a specific length k extracted from the input sequences. The edges of the graph represent connections between k-mers, where the length *k-1* suffix of one node matches the prefix of length *k-1* of another node. These connections are directed edges that move from the node representing the k-mer with the matching suffix to the node representing the k-mer with the matching prefix, as illustrated in a simple example in Fig. 2A. By combining linear paths (sequences of nodes connected to no more than two other nodes) into a single node called a unitig [31], [32], [33], the graphs can be compressed into cDBGs (Fig. 2B). This process resulted in a graph with the best possible resolution for specific areas, where long unitigs typically represented regions of the genome that were conserved among individuals, whereas shorter unitigs often indicated highly variable areas (Supplementary Fig. S1).

To construct cDBG, we used the DBGWAS tool [18], which involves several steps. First, each DBG was created using the GATB C+ + library [34], with contigs from each provided genome as input instead of using reads. Using contigs as starting points, we avoided the need to eliminate low-abundance k-mers. Next, a graph-traversal algorithm was used to condense the DBG and recognize any linear paths in the graph to produce unitigs in the cDBG. Additionally, during this stage, each k-mer index was assigned its corresponding unit index in cDBG. As highlighted in [18], the selection of the ideal k-mer length is a nuanced process that depends on several factors, including assembly quality, genome complexity, and the presence of repeats. As k increases, the defined genetic patterns (haplotypes) become more tailored to specific subgroups within a population. However, this specialization has a drawback in that it reduces the ability to identify connections between genetic variations and observable traits (genotype-to-phenotype associations). In contrast, low k values yield broadly connected non-specific patterns, and regions with repeats of at least k bases can cause complications by creating cycles in the DBGs. Given that a k-mer length of k = 31 demonstrated optimal performance in retrieving known markers of *P. aeruginosa* resistance to amikacin and levofloxacin in a previous study [18], we used this parameter to construct DBGs for this study.

### 3.2 Pan-genome construction

The representation of each strain in our analysis was based on a vector that captured the presence or absence of each unit in cDBG. This process involved utilization of the DBGWAS package, which was seamlessly integrated into the GATB C+ + library. First, a single DBG was created from the input genomes using the GATB C+ + library. Subsequently, a graph traversal algorithm within the DBGWAS-facilitated compaction of the DBG revealed linear paths that constituted the unitigs in the cDBG. During compaction, each k-mer index was intricately linked to its corresponding unit index within the cDBG. To extract unitig information for a given genome, GATB's Minimal Perfect Hash Function (MPHF) was employed to efficiently provide an index of the unitig associated with each k-mer. By leveraging the established correlation between k-mer and unitig indices, we accurately determined the unitigs present in each strain, thus forming the foundation for subsequent analyses.

Matrix M was used to store the unitig information for all input genomes. Each element in the matrix, denoted by M_m,n_, indicates whether the n_th_ unit is present (M_m,n_ = 1) or absent (M_m,n_ = 0) in the m_th_ genome. This enabled easy comparison and analysis of the presence or absence of specific proteins across different genomes. Furthermore, to minimize the risk of false positives from testing underpowered unitigs and to save computational time, we screened unitigs based on their frequency. If the frequency of a unitig across all strains was less than 1%, it was removed in the filtering process. A rarefaction curve was constructed to assess whether the genome set used in our study was sufficiently representative to capture most of the unique unitigs present in the population or if additional genomes were still needed to uncover more diversity. A curve that leveled off and approached an asymptote suggested that we had already observed most of the unitigs in the target population. Conversely, a curve exhibiting a steep increase indicated that additional genomes may be required to uncover additional unique unitigs.

To validate the effectiveness of our ML model, we constructed a pan-genome for the independent validation dataset by mapping all selected features back to each genome in the validation dataset using NCBI BLASTN (-evalue 1e-5 -max_target_seqs 1 -perc_identity 100), generating a presence/absence table for the validation dataset. This approach enabled us to verify the accuracy and reliability of our study and mitigate the potential effects of the curse of dimensionality.

### 3.3 Antibiotic selection for prediction

To obtain AMR phenotypes for each antibiotic, we extracted data from the “PATRIC_genomes_AMR.txt” file, provided by PATRIC [21]. For the present study, we restricted our analysis to strains annotated as either "resistant" or "susceptible.” To create an individual pan-genome for each antibiotic, data linking each antibiotic to its corresponding genome were gathered and incorporated into the pan-genome to build a machine learning prediction model. This study involves the creation of distinct AMR classifiers for various drugs that correspond to *P. aeruginosa*. For the analysis, only antibiotics that met certain requirements were included. Specifically, only drugs with a minimum of 300 entries for both resistant and susceptible strains and less than a five-fold difference between them were analyzed. These criteria aimed to prevent data imbalances and ensure an adequate number of samples for accurate model training while remaining consistent with established clinical guidelines [35]. Consequently, eight antibiotics (meropenem, amikacin, ceftazidime, ciprofloxacin, gentamicin, tobramycin, imipenem, and levofloxacin) were tested. Supplementary Table S2 presents the detailed counts of both resistant and susceptible strains corresponding to each of the selected antibiotics.

### 3.4 Feature selection

Feature selection is a crucial process when building a predictive model, where significant features that contribute to the outcome variable are identified and irrelevant or redundant features are eliminated to improve model performance, reduce overfitting, and increase interpretability [36], [37], [38]. In the present study, the eXtreme Gradient Boosting (XGBoost) algorithm was configured with specific parameters (such as objective = “binary:logistic”, importance_type = “gain,” max_depth = 6, and n_estimators = 500) to extract features (gene clusters) relevant to resistant or susceptible phenotypes. Subsequently, only the unitigs with importance values of > 0 were selected to build the ML model.

### 3.5 Classification algorithm

ML methods are becoming increasingly prominent in AMR research because of their capacity to manage high-dimensional features and their advantages in identifying intricate correlations among features and feature combinations to build effective predictive or prognostic models [38], [39], [40], [41]. The XGBoost-selected feature subset for each antibiotic was used to train the model. Next, we used the XGBoost-supervised ML model to conduct a binary classification of *P. aeruginosa* strains into either resistant or susceptible categories for each of the selected antibiotics. The XGBoost algorithms belong to the genre of ensemble learning techniques and fall under the category of boosting tree algorithms. This approach amalgamates predictions from numerous weak learners and progressively improves the model by addressing the errors made by previous trees. In other words, the algorithm collects predictions from diverse weak learners and selects the best model based on their combined votes. XGBoost was selected as the primary AMR predictor based on a meticulous comparative analysis. Various well-established ML models, including random forest (RF), AdaBoost, logistic regression (LR), support vector machine (SVM), and XGBoost, were trained and rigorously evaluated for their AMR predictive capabilities.

To evaluate the predictive outcomes, we adopted a ten-fold stratified cross-validation strategy. This required splitting the dataset into ten portions of equal size and ensuring that each component contained approximately the same number of target variable classes. The model was trained on nine parts and evaluated on the tenth, and the process was repeated ten times on different data portions to obtain an average performance metric. To facilitate comprehensive comparisons, we repeated this procedure ten times for each antibiotic, thereby obtaining robust average measurements. The scikit-learn (https://scikit-learn.org/) and XGBoost (https://pypi.org/project/xgboost/) packages were instrumental in facilitating the training and evaluation of all the models in this comprehensive analysis. Parameters for the models were set as follows: SVM with the linear kernel, C= 1.0; RF, LR, and AdaBoost with default settings; and the XGBoost classifier with parameters objective= “binary:logistic”, max_depth= 6, and n_estimators= 500. The decision to prioritize XGBoost was informed by a comprehensive assessment of its performance across a range of evaluation metrics, as detailed in Supplementary Table S3, which shows the mean values for each model across external and independent validation datasets.

### 3.6 Performance assessment and statistical analysis

The assessment of model performance incorporated a suite of well-established evaluation metrics, including accuracy, precision, recall, and the F1-score (the harmonic mean of precision and recall). Furthermore, we leveraged the area under the receiver operating characteristic (ROC) curve (AUROC, also called AUC) to assess the AMR classification process of our approach. During model training, we utilized three distinct feature sets: one comprising all unitigs, another containing unitigs with AMR genes (amr-gene-unitigs), and a third incorporating unitigs identified using our customized XGBoost-based approach (XGB-unitigs). The amr-gene-unitig feature subsets were derived through several steps. Initially, CD-HIT v4.6 (-c 0.95 -d 1) [42] was used to create gene clusters from the amino acid sequences of all strains. Next, we detected the protein sequences containing known AMR genes by querying the representative sequences of the gene clusters against the CARD database [43] using the accompanying Resistance Gene Identifier software (RGI v5.0.0). Subsequently, we utilized BLASTX with an e-value threshold of 0.01 to identify the presence of AMR proteins within AMR gene-associated unitigs. Finally, presence/absence tables for unitigs with AMR genes were constructed for all selected antibiotics. Detailed information on the number of samples used to train the model and the number of features within each feature set are provided in Supplementary Table S4. To discern the most effective feature set, the XGBoost classifier was consistently used for the three feature sets, which allowed for a robust comparison of the classifier performance across different feature sets. This approach ensured comprehensive evaluation of the effectiveness of the model in capturing AMR patterns across diverse genomic features. We also conducted rigorous comparisons using the Wilcoxon rank-sum tests. Statistical significance was denoted by *p*-value of < 0.001 (***), < 0.01 (**), or < 0.05 (*).

### 3.7 Exploration of the XGBoost-feature subset and its gene content

**Fig. 3:**
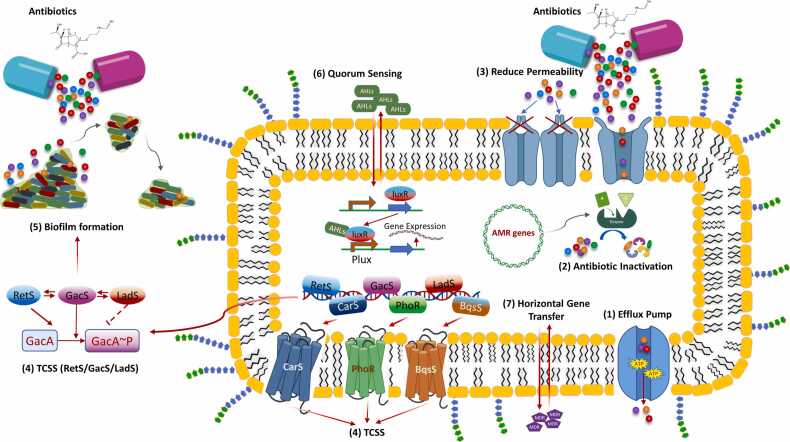
Mechanisms of AMR in *P. aeruginosa*. These mechanisms are classified into intrinsic, acquired, and adaptive resistance. Intrinsic resistance encompasses factors such as (1) efflux systems, reduced outer membrane permeability, and (2) antibiotic inactivation by various enzymes. Acquired antibiotic resistance involves resistance by mutations and acquisition of resistance genes (7). Mutations and acquisition of resistance genes occur through HGT. Adaptive antibiotic resistance includes (5) biofilm-mediated resistance. Virulence factors encompass (1) efflux systems, (4) two-component signaling systems (TCSSs), and (6) quorum sensing.

Over an extended period of evolution, bacteria have evolved several ancient genetic defence mechanisms that display considerable adaptability to counteract detrimental antibiotic substances. These mechanisms empower them to effectively react to a range of environmental dangers, including potential hazards such as antibiotics, chemicals, and antimicrobial peptides, all of which threaten their existence. The development of antibiotic resistance in *P. aeruginosa* is a multifaceted process. As shown in Fig. 3, some primary AMR strategies have been elucidated, including outer membrane permeability, efflux systems, antibiotic-deactivating enzymes, and biofilm formation.

In this study, we meticulously examined the functional significance of the unitigs selected using our approach, and their correlation with AMR. Functional gene annotations were extracted from the PATRIC database. We then traced the unitigs from the XGBoost feature subsets back to *P. aeruginosa* genomes using BLASTN to establish gene associations. These genes were systematically categorized based on their involvement in prevalent AMR mechanisms and bacterial defense and survival strategies to serve two main objectives: to provide insights into our model's predictions and to ensure comprehensive gene classification. A comprehensive classification of the genes identified by our model is illustrated in Fig. 3, and detailed explanations are provided in Table 1. Additionally, genes labeled as “hypothetical proteins” were considered, whereas those without clearly established connections or contributions to AMR mechanisms were classified as “uncertain AMR genes.”

**Table 1:** Summary and explanation of antimicrobial resistance (AMR) mechanisms in *Pseudomonas aeruginosa*.

AMR mechanism/Defense strategy	Explanation
Virulence factors	*P. aeruginosa* possesses an array of virulence factors that contribute to its ability to cause infections. Regulatory systems such as two-component signaling and quorum sensing enable the bacterium to detect environmental cues, including antibiotics, and modulate virulence factors and AMR genes. This coordination aids in developing resistance mechanisms[71], [76], [77], [78]. Essential virulence factors include elastase, exotoxin A, and rhamnolipids, in addition to proteins in the quorum sensing system. Recognizing these factors is vital for designing strategies with anti-virulence agents to combat drug resistance[71], [79], [80].
Reduction of outer membrane permeability	The bacterial outer membrane limits antibiotic entry, driving resistance[81], [82]. Two pathways are present: lipid-mediated for hydrophobic antibiotics and porins for hydrophilic antibiotics. The outer membrane composition affects bacterial sensitivity, and modifying it often leads to resistance. Understanding these mechanisms is crucial for effective antibiotic development[82], [83].
Antibiotic-inactivating enzymes	Antibiotic-inactivating enzymes alter antimicrobial molecules, making them ineffective. Beta-lactamases, such as those hydrolyzing beta-lactam antibiotics, are widespread examples. Diverse hydrolases, oxidoreductases, and transferases modify antibiotics through hydrolysis, redox reactions, or functional group transfer[84], [85]. Resistance to fluoroquinolones results from mutations in topoisomerases and gyrase[84], [86]. Other antibiotics encounter resistance through enzyme production and altered bacterial targets. These include classes such as trimethoprim, sulfonamides, aminoglycosides, chloramphenicol, and quinolones[85].
Acquired antibiotic resistance	Bacteria can acquire antibiotic resistance through two distinct mechanisms: acquisition of resistance genes through HGT, which imparts resistance to particular antibiotics, and adaptive mutations involving genetic changes that alter the affected target sites or metabolic processes affected by the drugs[84], [87].
Adaptive antibiotic resistance	Bacteria develop adaptive resistance to antibiotics by altering gene and protein expressions in response to various environmental cues[87], [88], [89]. In *P. aeruginosa*, biofilm formation is a common method for acquiring adaptive antibiotic resistance[51], [90], [91].

## 4 Results

### 4.1 Exploring the bacterial pan-genome

**Fig. 4:**
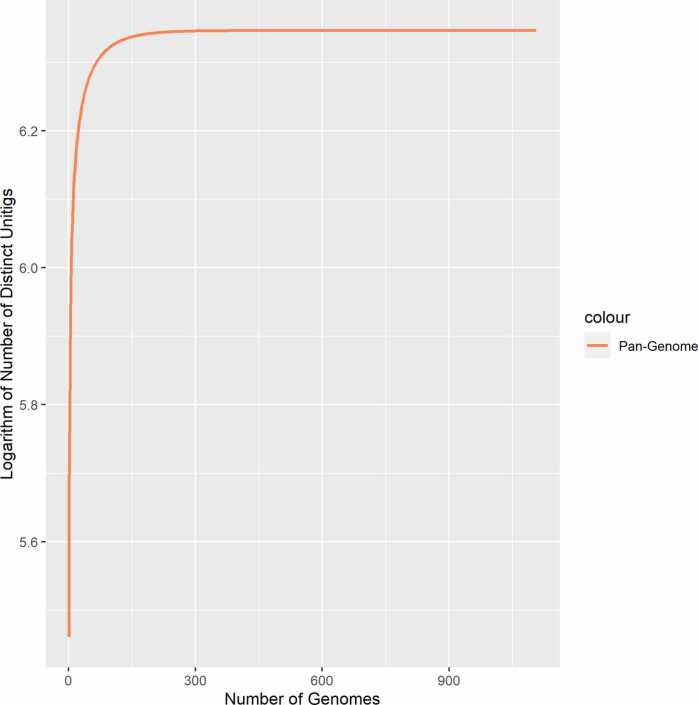
Pan-genome growth curve of *P. aeruginosa*. The X-axis displays the quantity of genomes (strains), whereas the Y-axis signifies the count of distinct unitigs.

Knowing whether a pan-genome is open or closed is important because it provides insightful information regarding the genetic diversity of a species and the number of genomes required to capture a complete set of genes within that species [44], [45]. Furthermore, it helps clarify the ability of a bacterium to acquire external DNA content and sheds light on the lifestyle characteristics that govern these abilities [46], [47]. In an open pan-genome scenario, the number of gene families increases as more genomes are included in the analysis, whereas in a closed pan-genome scenario, the number of gene families remains largely stable [46], [48]. After examining the genome quality, a pan-genome of 1107 *P. aeruginosa* strains was built for this investigation, and the unitig distribution of the pan-genome was analyzed by constructing a rarefaction curve, as shown in Fig. 4. The curve reached a saturation point, suggesting that *P. aeruginosa* possesses a closed pan-genome. The closed pan-genome of the species indicates that its genetic repertoire is relatively stable, with limited acquisition of novel genes across its population.

### 4.2 Unitig-based ML predictions of AMR phenotypes

**Fig. 5:**
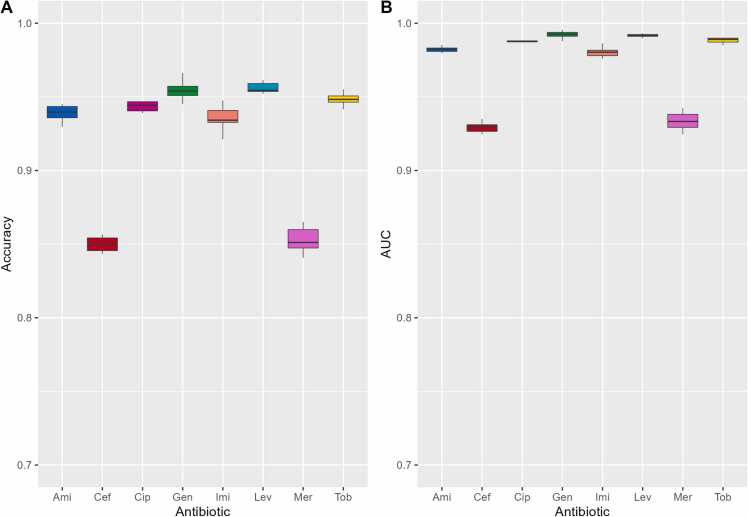
Performance evaluations of our AMR classifiers for various antibiotics in the training dataset. The left panel depicts the accuracy, and the right panel depicts AUC values.

A unitig presence/absence table for *P. aeruginosa* was created in relation to drug resistance phenotypes (refer to the “Methods” section for more details). To discern crucial unitigs within the presence/absence table for each antibiotic, we initially employed XGBoost to select the crucial features. Supplementary Table S5 shows the top 50 unitigs selected by the XGBoost algorithm, along with their corresponding importance scores. Subsequently, we implemented XGBoost classification models to classify the AMR phenotypes using a carefully selected feature subset selected by XGBoost in the previous step. Our results revealed that selecting relevant unitigs (features) using XGBoost led to satisfactory predictive performance (Table 2). The performance of the model with different antimicrobial agents is shown in Fig. 5. The results showed that the model performed well for most antimicrobial agents on the training dataset, as indicated by accuracies ranging 0.849–0.956 and AUC values of > 0.929.

**Table 2:** Performance evaluations of our approach on the training dataset. These results represent the mean performance achieved through repetition of a 10-fold cross-validation process.

Antimicrobial agent	Sensitivity	Specificity	F1-score	Accuracy	AUC^a^
Imipenem	0.945	0.925	0.935	0.935	0.980
Amikacin	0.749	0.980	0.936	0.939	0.982
Ciprofloxacin	0.939	0.948	0.943	0.943	0.988
Tobramycin	0.897	0.971	0.948	0.949	0.989
Levofloxacin	0.938	0.971	0.956	0.956	0.992
Gentamicin	0.902	0.975	0.954	0.955	0.992
Meropenem	0.874	0.825	0.853	0.853	0.934
Ceftazidime	0.818	0.875	0.849	0.850	0.929

a AUC, area under the receiver operating characteristic curve.

**Fig. 6:**
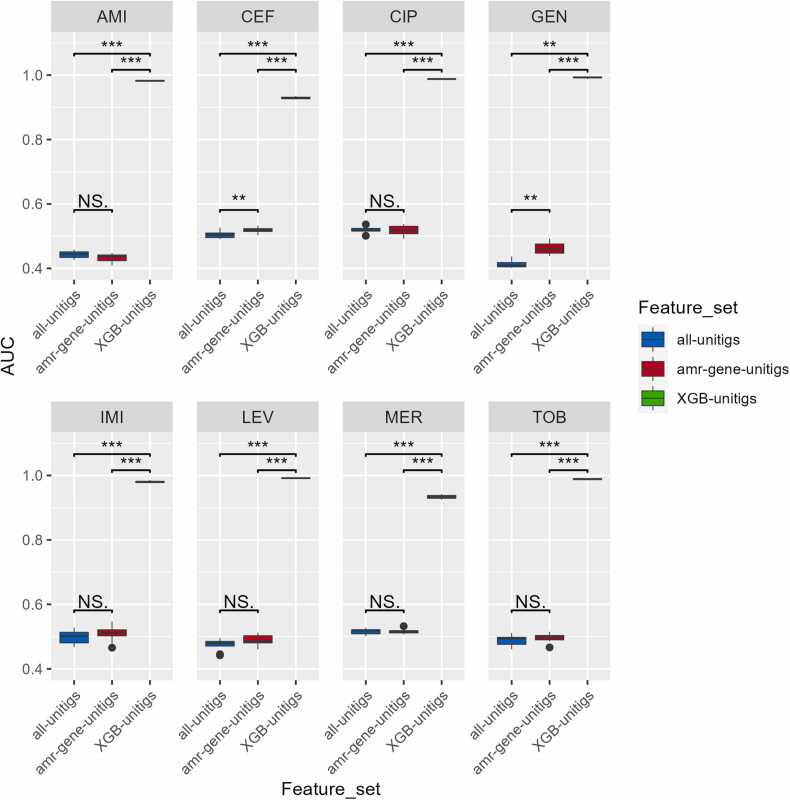
Comparative AMR classifier performance (AUC). This figure illustrates the performance evaluation of AMR classifiers using box-whisker plots that focus on the AUC. Performance is assessed across three distinct feature sets, with each plot corresponding to specific antibiotics. Outliers are indicated with solid circles. Significance levels: *p* < 0.001 (***), *p* < 0.01 (**), and *p* < 0.05 (*). Abbreviations for antibiotics: AMI, amikacin; CEF, ceftazidime; CIP, ciprofloxacin; GEN, gentamicin; IMI, imipenem; LEV, levofloxacin; MER, meropenem; and TOB, tobramycin.

To evaluate the effectiveness of feature selection algorithms in discerning AMR strains, we used the XGBoost algorithm to identify the most informative unitigs. Next, we compared the performance of the feature sets selected by XGBoost, referred to as XGB-unitigs, against both the complete set of unitigs and the unitigs containing AMR genes, termed amr-gene unitigs. As illustrated in Fig. 6, the XGB-unitig feature sets demonstrated superior performance compared with the other feature sets in terms of their proficiency in AMR classification. This underscores the efficacy of the proposed approach for an accurate AMR classification. In addition, the XGB-Unitig feature set exhibited AUC values above 0.929 for all medications, surpassing those of the other two feature sets by approximately 45%. Furthermore, it is important to highlight that for the majority of cases, the amr-gene-unitig feature set did not demonstrate notable differentiation from the all-unitig feature set in relation to AMR classification, except for ceftazidime and gentamicin. This observation implies that relying solely on known AMR genes is insufficient to capture the underlying AMR mechanisms associated with each antibiotic. Moreover, the proportion of AMR-related genes within the XGBoost feature subsets markedly surpassed that of the entire dataset, highlighting the effectiveness of our feature selection methodology in eliminating noise, while retaining crucial AMR indicators (Supplementary Fig. S2 and Supplementary Table S6).

Although our method exhibited outstanding accuracy on the training dataset, a key concern in ML approaches is their propensity to overfit. The validation results in Table 3, which were also evaluated using ten-fold cross-validation on an external validation dataset, summarize the performance of the extracted XGB-unitigs for four antibiotics: tobramycin and ciprofloxacin demonstrated strong predictive abilities with accuracies of > 0.788 and AUC values surpassing 0.830. This suggests that the model performed well in differentiating between samples susceptible and resistant to these antibiotics. Despite having slightly lower accuracy (a value of 0.73), ceftazidime still showed reasonable performance, with an AUC of 0.757. Additionally, the model appeared to face more challenges in the case of meropenem, with a comparatively low performance among the four antibiotics.

**Table 3:** Validation results of our model on the external validation dataset.

Antimicrobial agent	Sensitivity	Specificity	F1-score	Accuracy	AUC^a^
Tobramycin	0.715	0.894	0.835	0.837	0.830
Ciprofloxacin	0.800	0.775	0.786	0.788	0.831
Ceftazidime	0.752	0.722	0.734	0.737	0.757
Meropenem	0.804	0.400	0.668	0.678	0.665

a AUC, area under the receiver operating characteristic curve.

### 4.3 Functional analysis of selected unitig subsets

**Fig. 7:**
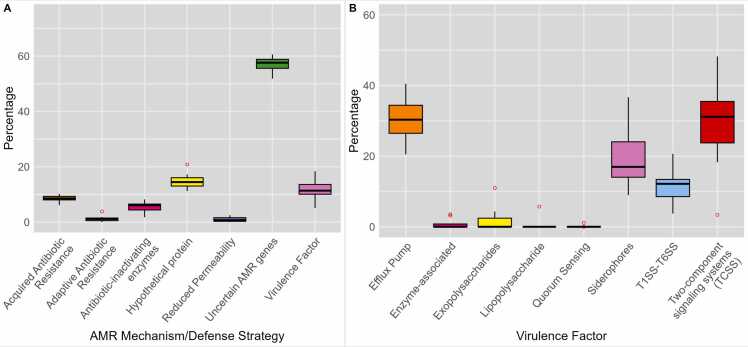
Gene content of XGBoost-selected feature subset. A. The percentage of genes identified using our approach and categorized based on common AMR mechanisms and bacterial defense and survival strategies observed in *P. aeruginosa*. B. Proportion of genes within the XGBoost-selected feature subset classified as virulence factors, accounting for diverse virulence factor subgroups.

To better understand the genes corresponding to the selected unitigs, we also examined and categorized them according to the AMR mechanisms or defense strategies commonly observed in *P. aeruginosa*. Fig. 7 illustrates the diverse AMR mechanisms and bacterial defense and survival strategies identified in the XGBoost feature subsets selected by our model, highlighting the involvement of multiple mechanisms in the development and spread of AMR phenotypes. Notably, the genes that had no clear relationship with AMR (categorized as ‘uncertain AMR genes’) accounted for approximately 58% of the identified genes. The second highest group consisted of hypothetical proteins (genes with unknown functions), which contributed a substantial proportion (approximately 17%). These findings suggest that the functions of numerous genes associated with AMR activity have not been previously characterized. In addition to these groups, virulence factors, acquired antibiotic resistance, and antibiotic-inactivating enzymes were the three notable gene groupings in the XGB-selected unit set at approximately 15% ( Fig. 7A). As shown in Fig. 7B, efflux pump systems and TCSSs were the two prominent groups, each accounting for approximately 36% of the genes among the various virulence factors, representing the highest gene counts compared to the other groups. Additionally, siderophores and T1SS–T6SS were also prominent virulence factor groups in the XGB-selected unit set (Supplementary Table S7). These findings indicate that *P. aeruginosa* may develop AMR through different strategies, especially through a wide range of AMR-related enzymes, acquisition of resistance genes, and TCSSs. The diversity of AMR mechanisms and bacterial defense and survival strategies that we discovered highlights the complexity of AMR development and the importance of considering multiple factors in combating AMR in *P. aeruginosa*.

### 4.4 Unveiling co-resistant genes and MDR mechanisms: a systematic comparison of selected unitig subsets

Multidrug-resistant *P. aeruginosa* (MDRPA) presents a substantial clinical quandary due to its ability to evade multiple antibiotics, including critical choices such as carbapenems. The prevalence of MDRPA is increasing notably, often showing resistance to three or more antimicrobial agents, with some strains displaying near-complete resistance to antibiotics [49], [50]. Resistance mechanisms involve intrinsic factors, such as efflux pumps, limited outer membrane permeability, and biofilms [51]. Genetic mutations can also contribute to enhanced resistance [52]. In light of the AMR of *P. aeruginosa*, novel strategies for treating AMR have been explored. These combination therapies integrate diverse antibiotics and innovative non-antibiotic agents such as bacteriophages and antimicrobial peptides [51]. In this study, we systematically compared all unitig sets for eight antibiotics, aiming to unveil gene insights and their links to AMR in *P. aeruginosa*, with specific emphasis on the genes that contribute to multidrug antibiotic resistance.

**Fig. 8:**
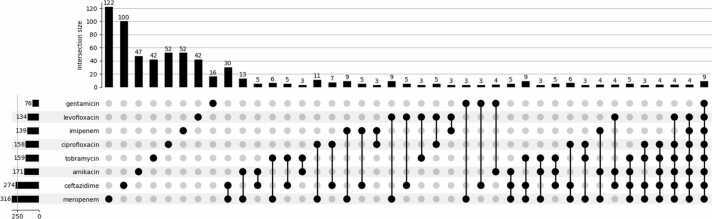
Antibiotic gene set intersections. This figure illustrates the intersection of eight sets of antibiotic genes. The intersection size represents the number of genes shared between multiple sets. For instance, an intersection of meropenem and ceftazidime with a value of 34 implies that there are 34 genes shared between these two antibiotics. The left lower panel represents the set size, which denotes the number of genes in each individual set. For example, the AMR gene set associated with meropenem and ceftazidime comprises 336 and 287 genes, respectively.

The analysis of gene patterns across various feature subsets of the eight antibiotics is presented in Fig. 8. Interestingly, nine co-resistance genes, excluding hypothetical proteins, were observed in all feature subsets. In addition, another nine genes were identified in conjunction with seven antibiotics, whereas another set of nine genes was shared among the six antibiotics (detailed information is provided in Supplementary Table S8). This intriguing pattern underscores the importance of these shared genes in AMR classification. This suggests a compelling potential for their involvement in co-resistance mechanisms within *P. aeruginosa*, highlighting promising associations that merit further investigation.

**Fig. 9:**
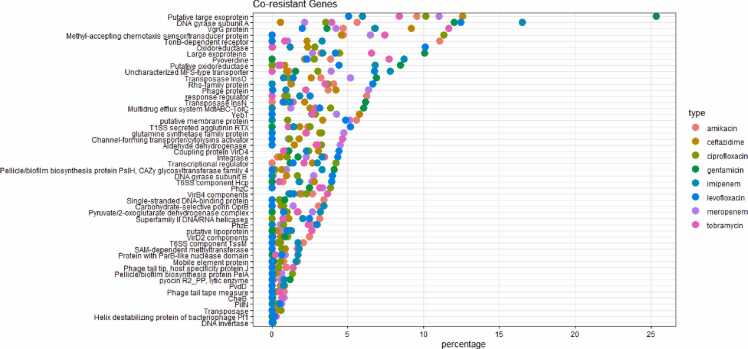
List of co-resistant genes that are shared across multiple antibiotics. Genes occurring across a higher number of antibiotics are positioned at the front of the list.

A notable prevalence of mobile genetic elements (MGEs) such as integrases, transposases, and other mobile elements, categorized under the ‘antibiotic-inactivating enzymes’ mechanism, among intersections of co-resistant genes, as illustrated in Figs. 9 and 7A, were also observed. Beyond MGEs, the T1SS–T6SS subgroup, which fell within the virulence factor category, emerged as the second most prominent subgroup frequently encountered in co-resistance gene compilation. Notably, despite the efflux system, TCSSs, and siderophores constituting the three largest subgroups within the virulence factor category, MGEs and T1SS–T6SS demonstrated pivotal roles in MDR progression, as shown in Fig. 7B.

MGEs, which encompass insertion sequences and transposons, play substantial roles in the distribution of genes associated with AMR in bacteria. These elements can relocate themselves along with linked resistance genes across diverse bacterial strains and species, thereby aiding the propagation of AMR [53]. For example, the transposase InsO was associated with the insertion sequence element IS911, but was not immediately linked to AMR in *P. aeruginosa*. However, insertion sequences such as IS911 can harbor passenger genes that propagate antibiotic resistance [54], [55]. Moreover, integrons, notably class 1 integrons, play a pivotal role in antibiotic resistance in *P. aeruginosa* [56]. These structures accommodate gene cassettes that bestow resistance to clinical antibiotics, allowing bacteria to acquire and develop resistance to multiple drugs, such as β-lactamases, fluoroquinolones, and carbapenems [56]. The integrase enzyme, which is governed by the *intI1* gene and is specific to class 1 integrons, critically integrates and reorganizes gene cassettes. This mechanism allows the bacterium to activate resistance genes in response to antibiotic exposure. Moreover, integron and integrase activities affect the bacterium's adaptive reaction to antibiotics, as exemplified by a study involving an adapted integron that heightened resistance progression [57].

Genes related to secretion systems include valine-glycine repeat protein G (*VgrG protein*), *VirB4* components, *Coupling protein VirD4, ATPase required for T-DNA transfer*. Notably, *VgrG protein* assumes a pivotal role in the T6SS, an intricate mechanism that enables bacteria to introduce toxic proteins into neighboring cells, thereby enhancing virulence and survival [58]. Although *VgrG protein* may not directly contribute to AMR, its amplification of the overall bacterial virulence and resilience is significant. Additionally, VirB4 is a key protein in the T4SS machinery [59]. VirB4 plays a pivotal role in the T4SS machinery [73] and facilitates effector protein translocation into host cells [60]. Although a direct link between the T4SS, VirB4, and *P. aeruginosa* AMR is lacking, the involvement of the T4SS in horizontal gene transfer (HGT) could facilitate AMR gene dissemination [61]. Another element, VirD4, functions as a coupling protein within the T4SS but has no direct *P. aeruginosa* AMR link [62]. However, its role in DNA transport [63] hints the potential facilitation of horizontal AMR gene transfer.

The remaining genes consisted of the ‘*Putative large exoprotein associated with heme utilization or adhesion from the ShlA/HecA/FhaA family*’ and the ‘*Large exoprotein involved in heme utilization or adhesion*.’ However, the association between these genes and AMR in *P. aeruginosa* remains unclear. Nonetheless, prior research has highlighted the significance of virulence factors, including diverse exoproteins, in pivotal roles such as bacterial adhesion, colonization, suppression of host immunity, and evasion mechanisms [64], [65], [66]. Further in-depth investigations are warranted to comprehensively understand the potential connections between these genes and AMR. Such studies hold promise for providing insights that bridge the gap between theory and clinical reality, thereby shedding light on the intricate relationships between these candidates and AMR.

## 5 Discussion

In the present study, we aimed to build a pan-genome and develop an explainable ML model to predict AMR phenotypes in *P. aeruginosa*. Our findings demonstrate that the presence/absence of unitig patterns extracted from cDBGs can effectively classify antibiotic-resistant pathogens, suggesting that the presence or absence patterns of these unitigs can serve as indicators to predict the resistance of bacterial pathogens to particular antibiotics. Notably, our ML model successfully classified AMR phenotypes, even without prior knowledge of the AMR mechanisms, allowing us to explore the full repertoire of genes and provide novel perspectives for understanding and addressing AMR in *P. aeruginosa*. By exploiting the power of ML and pan-genomic analyses, our research sheds light on the sophisticated associations between genomic variants and AMR, opening new perspectives for understanding and addressing AMR.

A closed pan-genome scenario was observed after rarefaction of 1107 *P. aeruginosa* strains, which showed that the gene repertoire of this species was relatively stable, with limited acquisition of novel genes across its population. The unitig pan-genome from the cDBGs was used to train the AMR predictor, leading to satisfactory performance. This suggests that the unitigs were able to capture the majority of the genetic information within the species, including the variable and flexible regions that make up the accessory genome. Indeed, our model exhibited robust AMR discriminative abilities for most of the tested drugs, as evidenced by high accuracies ranging from 0.849 to 0.956 and AUC values exceeding 0.929 on the training dataset. Additionally, an independent external dataset was utilized for model evaluation to minimize the risk of overfitting and ensure reliable predictions. In the independent validation dataset, our model demonstrated acceptable performance, with an accuracy and AUC value of approximately 0.77. These results support the strong classification power of the developed algorithm, which effectively classified AMR for several antimicrobial agents and made precise predictions. The slightly reduced performance of meropenem may be attributed to the presence of intricate or uncommon genetic variations associated with meropenem resistance. Further examination and potential expansion of the dataset with more varied meropenem-resistant strains can help enhance the performance of our model for this particular antibiotic.

Further analysis of the selected unitig sets indicated that approximately 58% of the genes did not have a clear relationship with AMR (excluding hypothetical proteins), and were categorized as “unclear AMR genes,” suggesting that there are still numerous uncharacterized genes associated with AMR activities. Approximately 16% of the genes were categorized as hypothetical proteins with unknown functions, emphasizing the need for further studies to elucidate their roles in AMR. Our research emphasizes the importance of enhancing our understanding beyond the established factors to yield a more complete understanding of AMR. The fact that we observed a diverse range of AMR mechanisms and defense strategies in *P. aeruginosa* such as ‘acquired antibiotic resistance, virulence factors, and the acquisition of resistance genes, is not surprising. This indicates the propensity of the pathogen to create more sophisticated AMR pathways that can compromise the effectiveness of multiple drugs. This pathogen is highly adaptable and possesses a variety of intrinsic and acquired resistance mechanisms against antibiotics. These mechanisms and strategies help to endure and flourish in the presence of various antimicrobial agents. As described in Table 1, the bacteria can utilize efflux pump systems to actively pump out antibiotics, produce beta-lactamases that hydrolyze beta-lactam antibiotics [67], [68], modify antibiotic targets, lessen the permeability of the bacterial outer membrane [69], generate protective biofilms [70], sense and respond to environmental stimuli [71], employ quorum sensing to modulate resistance genes [72], acquire resistance genes through HGT [73], and evolve resistance through adaptive mutations [74]. These diverse mechanisms enable *P. aeruginosa* to exhibit resistance to multiple types of antibiotics, making it difficult to treat the infections caused by this pathogen.

In alignment with these significant findings, our study also unveiled a comprehensive list of co-resistance genes that exhibited a significant presence across multiple drug categories, some of which spanned all tested antibiotics. Notably, our investigation identified MGEs as the predominant subgroup recurrently encountered within this compilation of co-resistance genes. This subgroup encompasses elements, such as phage proteins, integrases, and transposases, underscoring their pivotal role in facilitating the dissemination of AMR. Indeed, a previous study demonstrated the presence of MGEs harboring resistance genes such as *blaVIM-2* and *blaKPC-2* in MDRPA isolates [75]. Furthermore, a second notable group within the co-resistance gene intersections comprised genes associated with T4SSs, T6SSs, and T1SSs, which are typically categorized as virulence factors. These outcomes underscore the need for future investigations to delve deeper into the mechanisms governing co-resistance genes in *P. aeruginosa*, with a particular emphasis on elucidating the roles of MGEs and virulence factors associated with T1SS–T6SS. Such research is essential to devise innovative strategies to counteract the proliferation of MDR strains.

In addition to genes whose roles in causing AMR in *P. aeruginosa* have only been partly or fully revealed, there are other genes that may not directly induce AMR but are still involved in AMR development. For example, *VgrG protein*, an important component of the T6SS that plays a key role in toxic delivery, contributes to the virulence and pathogenicity of the bacterium. Moreover, the T4SS comprises various proteins, notably VirB4 and VirD4, both of which function as ATPases and supply the necessary energy for the transfer mechanism. The T4SS also plays a crucial role in the release of virulence factors that enhance bacterial pathogenicity. The virB/virD4 system facilitates the exchange of DNA and proteins between bacteria. More attention is needed to determine the link between the large exoproteins involved in heme utilization, adhesion, and AMR. Notably, current evidence is insufficient to conclude whether VgrG proteins, virB/virD4, or large exoproteins involved in heme utilization and adhesion are co-resistance genes or are directly linked to MDR. Further examination of the relationship between these genes and AMR could provide a more comprehensive understanding and assist in the development of targeted therapeutics to combat bacterial infections.

In conclusion, our study demonstrated the effectiveness of our method in constructing a unitig-centered pan-genome and developing an AMR predictor, allowing for systematic analysis across diverse bacterial genomes and not limited to only *P. aeruginosa*. By leveraging features derived from the pan-genome, our approach accurately identified significant AMR determinants and expanded the AMR gene repertoire, demonstrating its adaptability to different species. Our proposed method extends its utility beyond *P. aeruginosa*, which is emerging as a powerful tool for AMR research across a wide spectrum of bacterial genomes. We hope that this study will complement traditional AMR prediction approaches that rely on limited reference-based AMR factors, thereby leveraging our ability to improve effective tactics to combat AMR.

## CRediT authorship contribution statement

**Nguyen Quoc Khanh Le:** Conceptualization, Investigation, Supervision. **Yu-Wei Wu:** Conceptualization, Data curation, Funding acquisition, Project administration, Supervision, Writing – review & editing. **Duyen Thi Do:** Data curation, Formal analysis, Investigation, Methodology, Software, Validation, Visualization, Writing – original draft. **Ming-Ren Yang:** Formal analysis, Investigation, Software. **Tran Nam Son Vo:** Data curation, Software.

## Declaration of Competing Interest

The authors have no conflicts of interest to declare. All co-authors have seen and agree with the contents of the manuscript and there is no financial interest to report. We certify that the submission is original work and is not under review at any other publication.
