# Supplementary figures and images for: Unitig-centered pan-genome machine learning approach for predicting antibiotic resistance and discovering novel resistance genes in bacterial strains
Source: Comput Struct Biotechnol J. 2024 Apr 16;23:1864–76. doi: 10.1016/j.csbj.2024.04.035 (PMC11067008; doi:10.1016/j.csbj.2024.04.035)

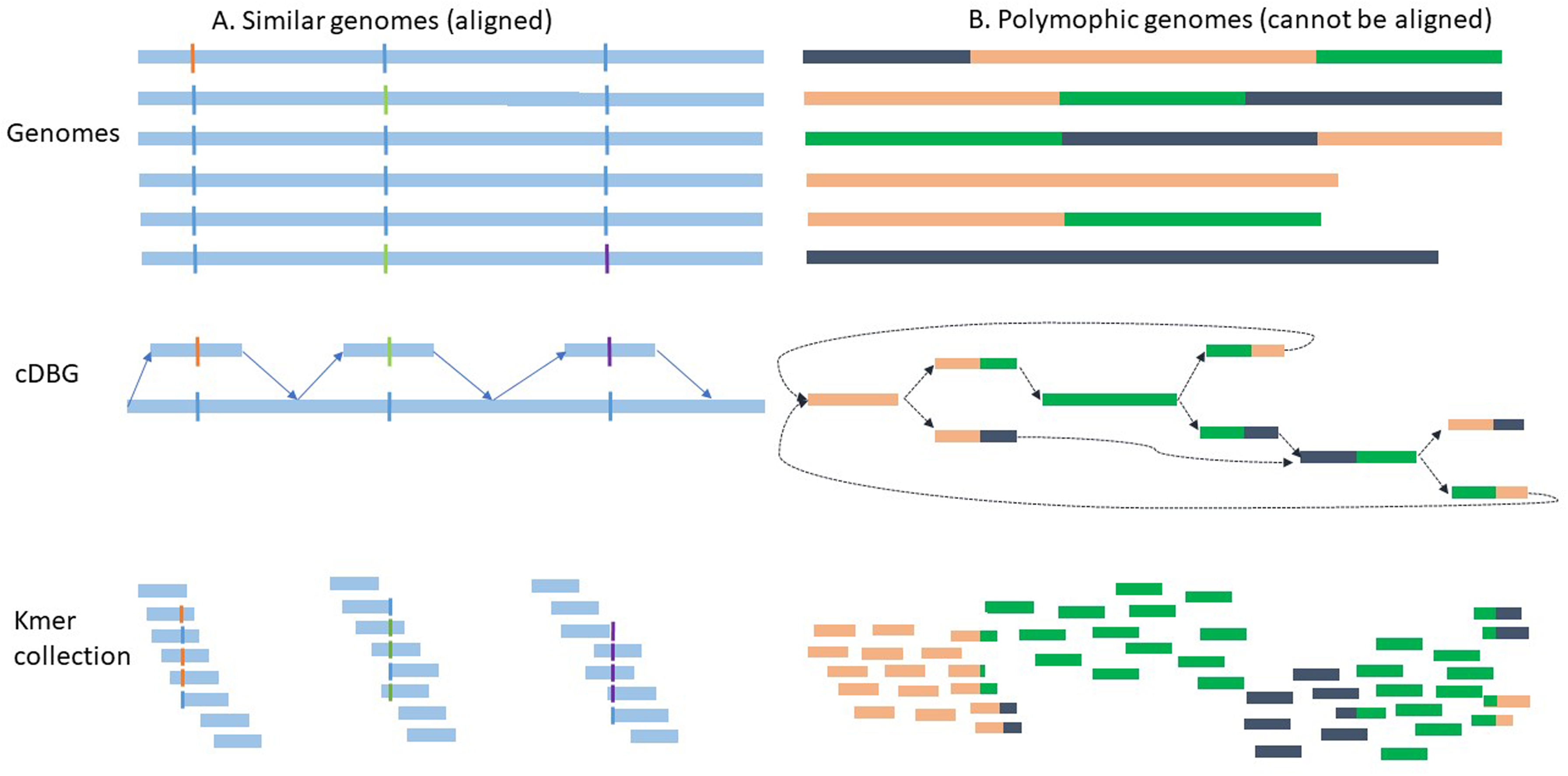

Supplement: Supplementary file 9 — Supplementary material [file mmc9.jpg]

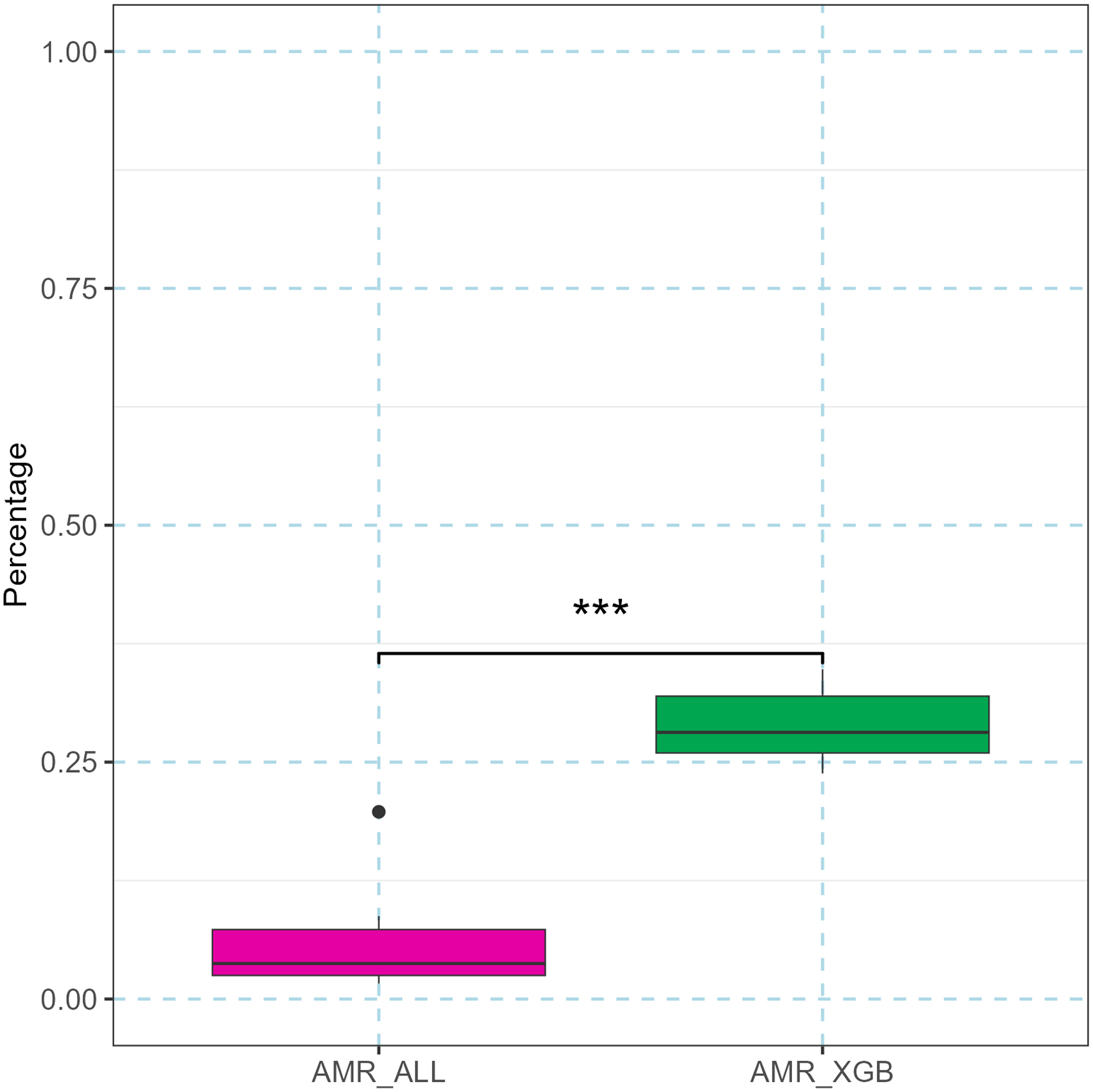

Supplement: Supplementary file 10 — Supplementary material [file mmc10.jpg]
